# Biodegradation of cefalexin by two bacteria strains from sewage sludge

**DOI:** 10.1098/rsos.220442

**Published:** 2023-01-11

**Authors:** Jichen Tian, Chong Chen, George Lartey-Young, Limin Ma

**Affiliations:** State Key Laboratory of Pollution Control and Resources Reuse, College of Environmental Science and Engineering, Tongji University, 1239 Siping Road, Shanghai 200092, People's Republic of China

**Keywords:** antibiotic pollution, cefalexin, biodegradation, degradation mechanism

## Abstract

Bioremediation has been used as an environmentally-friendly, energy-saving and efficient method for removing pollutants. However, there have been very few studies focusing on the specific antibiotic-degrading microorganisms in the activated sludge and their degradation mechanism. Two strains of cefalexin-degrading bacteria (*Rhizobium* sp. (CLX-2) and *Klebsiella* sp. (CLX-3)) were isolated from the activated sludge in this study. They were capable of rapidly eliminating over 99% of cefalexin at an initial concentration of 10 mg l^−1^ within 12 h. The exponential phase of cefalexin degradation happened a little earlier than that of bacterial growth. The first-order kinetic model could elucidate the biodegradation process of cefalexin. The optimized environmental temperature and pH values for rapid biodegradation by these two strains were found to be 30°C and 6.5–7, respectively. Furthermore, two major biodegradation metabolites of CLX-3, 7-amino-3-cephem-4-carboxylic acid and 2-hydroxy-3-phenyl pyrazine were identified using UHPLC-MS and the biodegradation pathway of cefalexin was proposed. Overall, the results showed that *Rhizobium* sp. (CLX-2) and *Klebsiella* sp. (CLX-3) could possibly be useful resources for antibiotic pollution remediation.

## 1. Introduction

Antibiotics play an essential role in human disease treatment, animal husbandry and aquaculture to prevent disease and promote the growth of animals [1,2]. However, with the extensive use and frequent detection of antibiotics in the environment, the antibiotic pollution has attracted increasing attention all over the world [3]. Since antibiotics cannot be fully metabolized by humans or animals, and wastewater treatment plants (WWTPs) cannot remove them completely, a large amount of them are being released into the environment [4]. It has been reported that antibiotics were found in different kinds of environmental media at concentrations up to μ g l^−1^ or μ g kg^−1^, including municipal wastewater [5], hospital wastewater [6], surface water [7], groundwater [8], soil and sediments [9].

**Figure 1.**
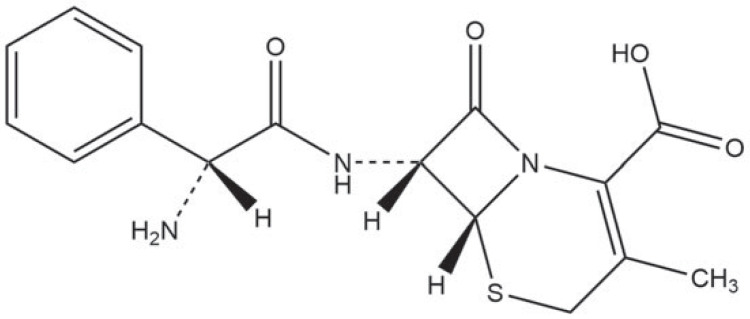
The molecular structure of cefalexin (CAS Number: 15686-71-2).

Cefalexin (5-Thia-1-azabicyclo4.2.0oct-2-ene-2-carboxylic acid, 7-(2R)-aminophenylacetylamino-3-methyl-8-oxo-, monohydrate, (6R,7R)-, figure 1), a first-generation broad-spectrum *ß*-lactam antibiotic, is prescribed in large quantities and used to treat infectious diseases in humans and animals [10,11]. In China, the annual usage of cefalexin was estimated to be 2670 tons in 2013 [1]. The residue of cefalexin in various environmental media suggests that the conventional wastewater treatment processes are unable to remove it thoroughly. Its persistence in the environment may contribute to the accumulation of antibiotic resistance genes (ARGs) and thus bring challenges to disease prevention [12]. Watkinson *et al*. [13] reported that cefalexin concentration could be up to 5600 ng l^−1^ in the influent of a wastewater treatment plant in Australia. In another study, the cefalexin concentration was found to be 170–5070 ng l^−1^ in sewage effluent and 6.1–493 ng l^−1^ in receiving seawater in the Hong Kong metropolitan area [14]. Therefore, it is vital to develop a method to remove the residual cefalexin more efficiently.

Compared with biodegradation, chemical treatments, especially advanced oxidation processes (AOPs) for cefalexin removal, have been studied more frequently [15–17]. But from the perspective of energy-saving, high efficiency and reducing secondary pollution, biodegradation is a practical method of antibiotic pollution remediation, which is gaining increasing attention worldwide [18]. Activated sludge is widely used for the treatment of wastewater contaminated with antibiotics in biological systems [11]. A few studies have been conducted to investigate the isolation of specific antibiotic-degrading microorganisms (mostly bacteria) and their degradation capability. For example, Pan *et al*. [19] found that a thermophilic bacterium strain, *Thermus thermophilus* C419, was able to remove more than 57% of ciprofloxacin at an initial concentration of 5 mg l^−1^ after 5 days of incubation. Shao *et al*. [20] isolated a novel oxytetracycline-degrading strain, *Ochrobactrum* sp. KSS10, which could achieve efficient oxytetracycline degradation and nitrogen removal at the same time. However, only two Pseudomonas strains and one Shewanella strain have been isolated and found to be capable of degrading cefalexin [21,22]. There is a need to find new types of cefalexin-degrading bacteria that have the potential to degrade cefalexin more efficiently from the local environment.

To our knowledge, very few studies have focused on the diversity of cefalexin-degrading bacteria, effects of environmental factors on biodegradation as well as characteristics and mechanism of cefalexin biodegradation. In this study, we aimed to isolate and identify different kinds of bacterial strains that were capable of degrading cefalexin and examine their growth and degradation characteristics. In addition, the effects of environmental factors (e.g. temperature, pH, inoculation dosage and initial cefalexin concentration) on degradation efficiency were examined and major metabolites during cefalexin biodegradation were identified for investigation of degradation mechanisms. This work could provide a practical tool for bioremediation of cefalexin pollution and insight into the biodegradation characteristics and mechanism of cefalexin by novel strains identified as cefalexin-degrading.

## 2. Material and methods

### 2.1. Chemicals and media

The activated sludge was sampled from Dazhong Jiading wastewater treatment plant (Shanghai, China). This sewage treatment plant is mainly responsible for collecting and treating the domestic sewage of the surrounding urban residents. Cefalexin (98.5%) was purchased from Yuanye Biotechnology Co. Ltd. (Shanghai, China). All the other chemicals (AR grade) were obtained from Sinopharm Chemical Reagent Co. Ltd. (Beijing, China).

The nutrient ager (NA) plates which were used for screening and isolation of cefalexin-degrading strains consisted of tryptone (10 g l^−1^), beef powder (3 g l^−1^), NaCl (5 g l^−1^) and agar powder (18 g l^−1^) in Milli-Q water. The Luria-Bertani (LB) media used for culturing and collecting bacteria was composed of tryptone (10 g l^−1^), NaCl (10 g l^−1^) and yeast extract (5 g l^−1^) in Milli-Q water. The mineral salt medium (MSM) used for enrichment of degrading strains and investigation of cefalexin degradation consisted of K_2_HPO_4_ (1.5 g l^−1^), KH_2_PO_4_ (0.5 g l^−1^), NaCl (1 g l^−1^), NH_4_Cl (1.5 g l^−1^), MgSO_4_·7H_2_O (0.2 g l^−1^), FeSO_4_·7H_2_O (3 mg l^−1^), ZnSO_4_·7H_2_O (4.5 mg l^−1^) and C_6_H_12_O_6_ (4 g l^−1^) as an extra carbon source in Milli-Q water. The stock solution of trace elements was prepared in advance and diluted while making the media. All media were adjusted to pH 7, and sterilized at 121°C for 20 min prior to use.

### 2.2. Enrichment and isolation of cefalexin-resistant strains

A preliminary test was first carried out. The activated sludge (1 ml) was inoculated into a 250-ml flask containing 100 ml of MSM with 10 mg l^−1^ of cefalexin to prove the degradation capability of the activated sludge on cefalexin, and the sterilized sludge (1 ml) was used as control.

**Figure 2.**
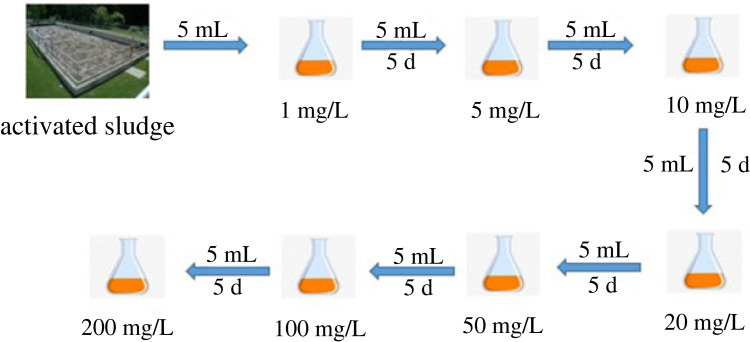
Process of enrichment of cefalexin-resistant strains.

For enriching bacterial strains with cefalexin resistance, a 5 ml activated sludge sample was inoculated into a 250-ml flask containing 100 ml of MSM with 1 mg l^−1^ of cefalexin and cultured by shaking inoculum at 150 rpm for 5 days at 30°C. This process was carried out under natural ventilation. Subsequently, 5 ml of the suspension obtained above was transferred into a new 250-ml flask containing 100 ml of MSM with a gradually increasing cefalexin concentration and also cultured by shaking inoculum at 150 rpm for 5 days at 30°C under natural ventilation. This process was repeated 6 times, and the concentrations of cefalexin in the 6 batches were 5, 10, 20, 50, 100 and 200 mg l^−1^ in turn (figure 2). Finally, 1 ml of the culture which was incubated with an initial cefalexin concentration of 200 mg l^−1^ for 5 days was serially diluted with sterile water and spread on NA plates. Different colonies with various morphological characteristics were isolated by streak-plating on NA plates and stored for further examination of their degradation capability on cefalexin and identification.

### 2.3. Identification of degrading strains

The identification of isolated degrading strains was based on 16S rDNA gene sequence analysis. The genomic DNA of the strains was extracted using the bacterial genomic DNA extraction kit (Takara, Japan). Then a polymerase chain reaction (PCR) was conducted for the amplification of bacterial 16S rDNA in the following condition: a denaturation step at 95°C for 5 min, followed by 35 amplification cycles of 95°C for 30 s, 58°C for 30 s, 72°C for 90 s and a final extension at 72°C for 7 min. The primers used for PCR were 27F (5-AGAGTTTGATCCTGGCTCAG-3) as the forward primer and 1492R (5-CTACGGCTACCTTGTTACGA-3) as the reverse primer. An agarose electrophoresis was performed to check the purity of the PCR products, which were then sequenced by Personalbio Co. Ltd. (Shanghai, China).

The 16S rDNA sequences of the determined cefalexin-degrading strains were compared against the available DNA sequences on GenBank using the basic local alignment search tool (BLAST) (www.ncbi.nlm.nih.gov/BLAST). The sequences were then deposited on GenBank with corresponding accession numbers. Phylogenetic trees of the degrading strains and closely related strains were constructed by the neighbour-joining method with MEGA 7.0 software.

### 2.4. Biodegradation of cefalexin and effects of environmental factors

Bacterial strains were first cultured in LB media under 30°C and 160 rpm for 24 h for quick proliferation. Then the bacterial cells were collected by centrifugation at 4°C and 5000 rpm for 10 min. After being washed by sterile phosphate buffer solution twice, the cells were resuspended in sterile water to reach an ultimate OD_600_ of 1.0. Then 1 ml of the bacterial suspension was added into the 250-ml flasks containing 100 ml of MSM (pH = 7) with an initial cefalexin concentration at 10 mg l^−1^. The culture was incubated under 30°C, 160 rpm for 48 h and the cefalexin concentration was determined within 0, 1, 2, 3, 4, 5, 6, 7, 8, 9, 10, 11, 12, 14, 24, 26, 28, 30, 32, 34, 46, 48 h intervals for studying the biodegradation characteristics of cefalexin by degrading strains. The biodegradation characteristics of cefalexin by *Klebsiella* sp. (CLX-3) from 14 to 48 h was not shown in Section 3. The sampling process was carried out under natural ventilation.

The process of cefalexin biodegradation was fitted with the first-order kinetic model. The equation of the model is expressed as equation (2.1) and the half-life is calculated as equation (2.2) [23,24]:

2.1 $$\ln\frac{C_{t}}{C_{0}}=\;-kt\;$$

and

2.2 $$T_{1/2}=\frac{\ln2}{k}$$

where *C_t_* is the concentration of cefalexin at time *t*, *C*_0_ is the initial cefalexin concentration, *k* is the degradation rate constant (*h*^−1^), *T*_1/2_ is the half-life of cefalexin.

Only one variable (e.g. temperature, pH, inoculation dosage and initial cefalexin concentration) was allowed among the treatments for determining the effects of each environmental factor on cefalexin degradation efficiency by bacterial strains. The impacts of different pH (5–9) under 30°C and different temperatures (20–40°C) under pH = 7 were investigated while the initial cefalexin concentration was set at 10 mg l^−1^ and the inoculation dosage was 1% (v/v). The effect of inoculation dosage (1%−10%, v/v) on degradation efficiency of 10 mg l^−1^ of cefalexin was studied under 30°C, and pH = 7. Similarly, the influence of various initial cefalexin concentrations (5–100 mg l^−1^) was studied under the condition of 30°C, pH = 7 with an inoculation dosage of 1% (v/v).

### 2.5. Pretreatment of cefalexin and its metabolites

A solid-phase extraction (SPE) was conducted using Oasis HLB extraction cartridges (Waters, MA, USA) for purification of the metabolites during cefalexin degradation. Briefly, 10 ml of the culture at 5 h and 24 h of cefalexin biodegradation by CLX-3 was centrifuged at 5000 rpm, 4°C for 10 min, then the supernatant was adjusted to pH = 5 and filtered by aqueous phase filter membranes. 5 ml of methanol and 5 ml of water were used for precondition of the extraction cartridges. The filtered samples were then loaded onto cartridges and the flow rate was controlled at less than 3 ml min^−1^. 5 ml of Milli-Q water was used to remove the inorganic matters attached to the HLB cartridges, and the water was discarded. 5 ml of methanol was used for final elution of the target compounds, and the eluate was collected in a separate brown bottle. Subsequently, the collected sample was concentrated by nitrogen blowing, diluted to a final volume of 1 ml with Milli-Q water and stored in a 2-ml brown vial for later determination.

### 2.6. Identification and quantification of cefalexin and its metabolites

The determination of cefalexin concentration was performed by high-performance liquid chromatography (Shimadzu, Japan). Prior to detection, 1.5 ml of suspension in MSM was centrifuged at 5000 rpm, 4°C for 10 min. The supernatant was then filtered by a 0.22 µm aqueous phase filter membrane and stored in brown vials for later use. A 10 µl aliquot of the sample was injected into an Athena C18 column (5 µm, 4.6 mm × 250 mm, Anpel Inc., Shanghai, China). The mobile phases used were water containing 0.5% of acetic acid/methanol (75 : 25) at a flow rate of 1.0 ml min^−1^. The column temperature and wavelength were set at 30°C and 254 nm, respectively. A standard curve of cefalexin concentration based on the external standard method was established.

For identification and determination of metabolites, an ultra high-performance liquid chromatography-mass spectrometer (UHPLC-MS, Thermo Fisher, MA, USA) was used to separate the potential intermediates during biodegradation. 10 µl of sample was injected into a CNW C18-WP column (3 µm, 150 mm × 2.1mm, Anpel Inc., Shanghai, China). The mobile phases were water containing 0.05% acetic acid /methanol and the gradient elution procedure was set as follows: 0–3 min, 20% methanol; 3–12 min, 20%−40% methanol; 12–15 min, 40% methanol; 15–20 min, 40%−20% methanol; 20–25 min, 20% methanol. The column temperature, injection volume and flow rate were 30°C, 20 ml and 0.3 ml min^−1^, respectively. The MS parameters were set as follows: electron spray ionization; capillary voltage: 4000 V; nebulizer pressure: 35 psi; nebulizer temperature: 300°C. Mass spectra were collected in the range of 150–500 *m*/*z* by full scan mode.

### 2.7. Determination of bacterial growth

The optical density values at a wavelength of 600 nm (OD_600_) were tested at the same interval as cefalexin concentration with a UV-2365 ultraviolet spectrophotometer (Unicosh Inc., Shanghai, China) for measurement of bacterial growth according to the method established by previous studies [25].

### 2.8. Statistical analysis

SPSS 22.0 software (SPSS, Chicago, USA) was used for comparing the differences between treatments at *α* = 0.05 via one-way analysis of variance (ANOVA) and subsequent least significant difference (LSD) comparison tests. Values with a *p* < 0.05 are considered significantly different.

## 3. Results and discussion

### 3.1. Isolation and identification of degrading strains

**Figure 3.**
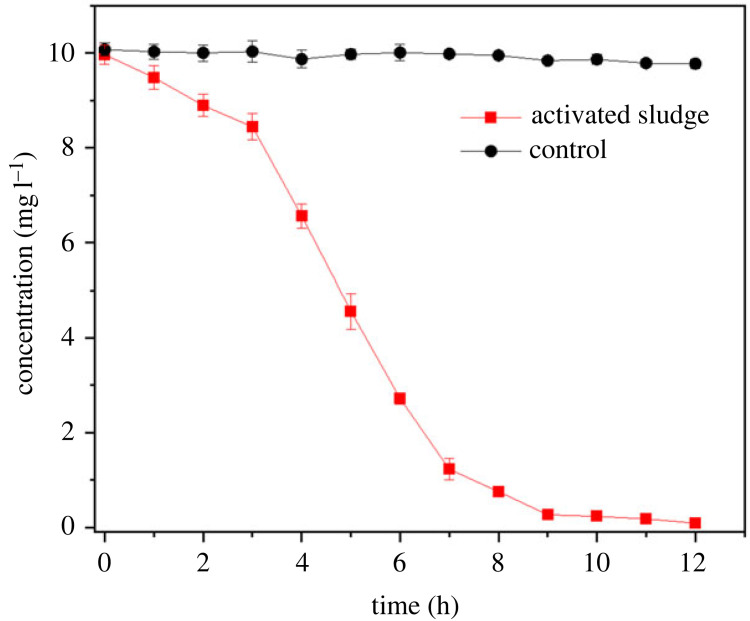
Elimination of cefalexin by activated sludge.

Media inoculated with sterilized sludge were used as a control for the examination of the degrading capability of the activated sludge from the WWTP. As indicated by the result in figure 3, there was no significant decline in cefalexin concentration while being treated with sterile sludge which has no bacterial activity, whereas the activated sludge treatment group efficiently removed cefalexin, whose concentration dropped from an initial value of 9.97 mg l^−1^ to 0.09 mg l^−1^, illustrating that activated sludge had an excellent ability to reduce this antibiotic in WWTPs, which has also been proven by a previous study [23]. Thus, it is necessary to determine the specific kinds of bacteria that are able to remove cefalexin efficiently.

A total of four strains with different morphological characteristics that could grow on NA plates containing 50 mg l^−1^ of cefalexin were isolated and purified using the streak plate method and their degradation rate on 10 mg l^−1^ of cefalexin within 24 h was detected (table 1). The results demonstrated that these strains were capable of eliminating cefalexin at significantly different degradation rates. Among the degrading strains, CLX-2 and CLX-3 were found to be able to remove more than 95% of cefalexin during this period, whose degrading efficiencies were much higher than those of the other two strains (CLX 4 and CLX 5), therefore two such strains deserve further identification and investigation for their degradation characteristics and mechanisms.

**Table 1.** Comparison of cefalexin degradation efficiency by different strains within 24 h. Note: different lowercase letters indicate significant differences (*p* < 0.05) among treatments.

strain	degradation rate (%)	standard deviation (%)
CLX-2	98.81 a	0.04
CLX-3	100.00 a	0.00
CLX-4	16.57 c	2.53
CLX-5	29.66 b	2.77

**Figure 4.**
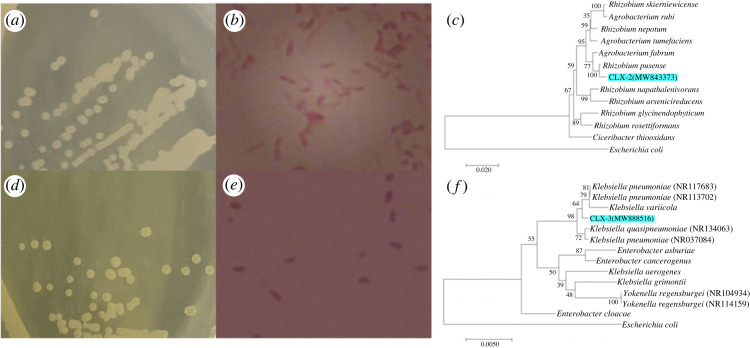
Microorganism morphology and phylogenetic tree of (*a–c*) CLX-2 and (*d–f*) CLX-3.

The colonial morphology of CLX-2 could be described as follows: white, opaque, circular and linearly arranged rods (figure 4*a*). The colonial morphology of CLX-3 was observed as yellowish white, opaque, circular and dull circles (figure 4*d*). The morphological features of the two colonies suggested that both CLX-2 and CLX-3 were Gram-negative bacteria (figure 4*b,e*). The 16S rDNA of CLX-2 and CLX-3 were amplified and sequenced. The sequences were submitted to GenBank with accession numbers of MW843373 and MW888516, respectively. According to the BLAST results, the 16 s rDNA sequence of CLX-2 had the highest similarity with that of *Rhizobium pusense* NRCPB10 (99.41%) while the 16 s rDNA sequence of CLX-3 had the highest similarity with that of *Klebsiella pneumoniae* DSM 30104 (99.64%). Therefore, these two strains were identified as *Rhizobium* sp. and *Klebsiella* sp., respectively. Phylogenetic trees were constructed with a group of strains that had a high similarity with these two strains using the neighbour-joining (NJ) method with MEGA 7.0 (figure 4*c,f*).

*Rhizobium* is an important kind of probiotic, which often lives in symbiosis with the roots of legumes and assists legumes in nitrogen fixation to promote the growth of plants [26]. So far, no report regarding the biodegradation of cefalexin by *Rhizobium* sp. has been found. However, this kind of bacteria has been proven to be able to degrade pollutants such as pesticides and PAHs [27–29]. A *Klebsiella* strain capable of degrading tetracycline and removing nitrate simultaneously was isolated from municipal sludge by Shao *et al*. [30], indicating that *Klebsiella* is a great candidate for both antibiotic pollution remediation and biological removal of nitrate [31]. To our knowledge, this manuscript first reported the cefalexin biodegradation by *Klebsiella*. The degradation characteristics and mechanism of the two strains thus deserve further investigation.

### 3.2. Bacterial growth and cefalexin degradation characteristics

**Figure 5.**
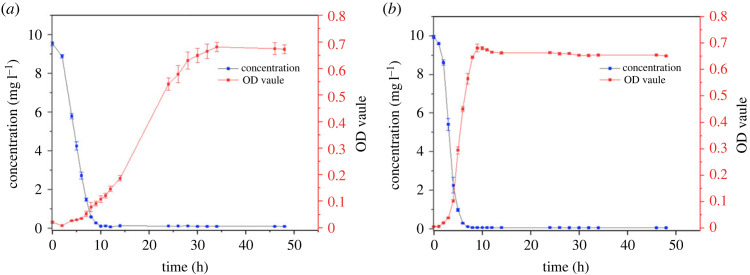
Bacterial growth and cefalexin degradation by (*a*) *Rhizobium* sp. (CLX-2) and (*b*) *Klebsiella* sp. (CLX-3).

The OD_600_ value and residual cefalexin concentration were measured at the same time interval for the determination of bacterial growth and cefalexin degradation characteristics of both strains (figure 5). According to the remaining cefalexin concentration during the degradation process, *Rhizobium* sp. (CLX-2) could degrade about 71% of cefalexin at an initial concentration of approximately 10 mg l^−1^ in 6 h while the degradation rate of cefalexin by *Klebsiella* sp. (CLX-3) under the same conditions reached more than 97%, demonstrating the higher cefalexin degradation capability of *Klebsiella* sp. (CLX-3). Both strains degraded more than 99% of cefalexin within 12 h. Lin *et al*. [21] isolated two cefalexin-degrading *Pseudomonas* strains, the biodegradation results illustrated that *Pseudomonas* sp. CE22 had a relatively higher cefalexin removal efficiency of over 90% in 24 h. Here in this study, two novel strains with much higher cefalexin-degrading efficiencies were isolated and characterized. Compared with other kinds of antibiotics, this study also illustrated the higher biodegradability of cefalexin indicated by its generally higher removal efficiency by microorganisms in 12 h [20,32–34].

The process of cefalexin biodegradation was fitted with first order kinetic model (table 2). The linear fitting results showed that the first order kinetic model could properly describe cefalexin biodegradation by both strains, especially *Klebsiella* sp. (CLX-3) (*R*^2^ = 0.96). The linear fitting results were in agreement with a previous study on biodegradation of several pharmaceuticals including cefalexin, which concluded that the biodegradation of these antibiotics followed first order kinetics [23]. The relatively lower *R*^2^ value for biodegradation may be attributed to the presence of the lag phase before the quick decline of residual cefalexin. Overall, the results indicated that cefalexin biodegradation is a concentration-limiting process and could be stimulated by the first order kinetic model, though some modification for the reaction model may be further needed for better description of the process.

**Table 2.** First-order kinetic model of cefalexin degradation by CLX-2 and CLX-3.

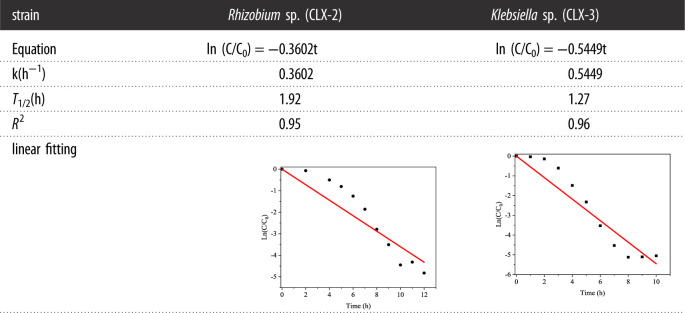

### 3.3. Optimization of environmental conditions

**Figure 6.**
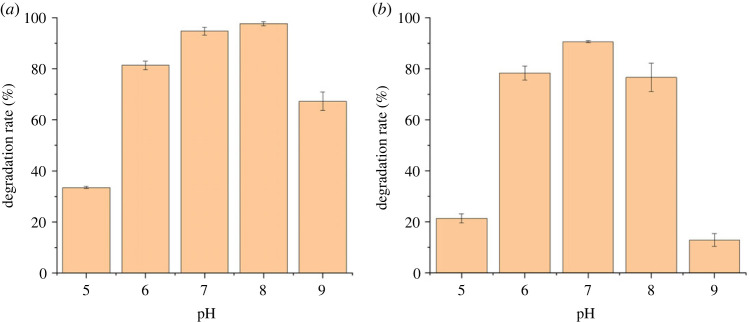
Effects of pH of the mineral salt medium (MSM) on degradation efficiencies of cefalexin by strain (*a*) CLX-2 and (*b*) CLX-3 for 12 h.

**Figure 7.**
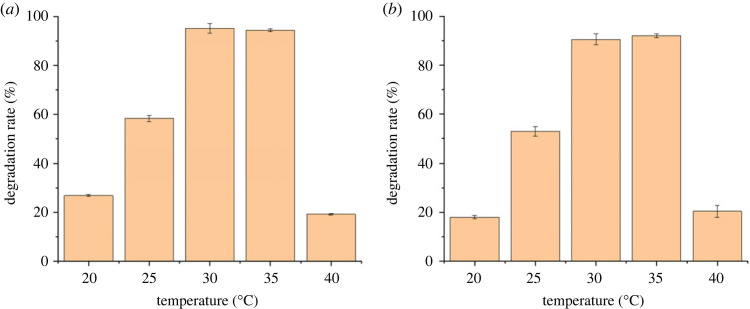
Effects of temperature of the mineral salt medium (MSM) on degradation efficiencies of cefalexin by strain (*a*) CLX-2 and (*b*) CLX-3 for 12 h.

pH is one of the key factors determining the bioreaction process and degradation rate of pollutants [35]. The results of cefalexin degradation under different pH conditions suggested that pH value had a significant influence on the degradation of cefalexin by *Rhizobium* sp. and *Klebsiella* sp. (figure 6). The optimal pH condition that achieved the highest removal of cefalexin is almost the same for the two strains (pH 7–8 for CLX-2 and pH 7 for CLX-3). Compared to the removal rate under highly acidic conditions (pH 5), CLX-2 performed better when cultured under highly basic conditions (pH 9). On the contrary, CLX-3 had a relatively higher degradation capability under acidic conditions. This phenomenon further indicated that *Rhizobium* sp. and *Klebsiella* sp. could survive well in the environment with pH 6–8. The optimum pH for *Rhizobium* sp. was 7–8, and it also showed certain acid and alkaline resistance compared with *Klebsiella* sp., for which the optimum pH was 7. Temperature could have a great impact on the process of biodegradation by influencing the activity of functional enzymes [36]. The results suggested that temperature had a similar influence on the degradation of cefalexin by two strains (figure 7). The removal of cefalexin was inhibited by either high (40°C) or low (20°C) temperature and the optimized temperatures for rapid cefalexin degradation by the two strains were both found to range from 30 to 35°C. The temperatures from 30 to 35°C were also consistent with the optimum temperature range for the growth of most microorganisms.

**Figure 8.**
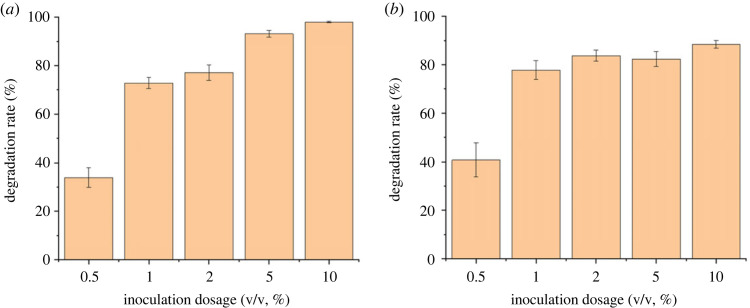
Effects of inoculation dosage in the mineral salt medium (MSM) on degradation efficiencies of cefalexin by strain (*a*) CLX-2 and (*b*) CLX-3 for 12 h.

The effect of inoculation dosage on cefalexin degradation by two strains was also significant. The removal rate of cefalexin rose with the increasing inoculation dosage and it can be seen that CLX-2 was more sensitive to the change in inoculation dosage, indicated by its significantly different degradation rate under the inoculation dosage of 2%, 5% and 10% (figure 8). However, the degradation rate of cefalexin by CLX-3 did not exhibit an obvious increase while the inoculation dosage rose from 2% to 10%. The inoculation dosage was also a key factor affecting the growth of *Rhizobium* sp. and *Klebsiella* sp. *Rhizobium* sp. could develop better with an increasing inoculation dosage from 1% to 10%, and *Klebsiella* sp. was able to develop well with an inoculation dosage of more than 1%. The growth trends for both strains were significantly restricted with an inoculation dosage of less than 1%.

**Figure 9.**
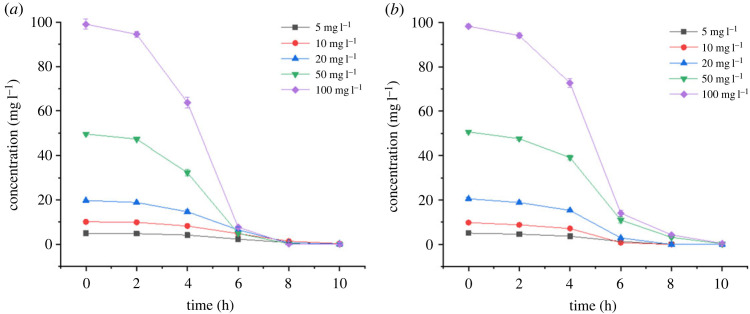
Effects of the initial concentrations of cefalexin in the mineral salt medium (MSM) on degradation efficiencies by strain (*a*) CLX-2 and (*b*) CLX-3 for 12 h.

In previous studies regarding the cefalexin degradation ability of bacterial strains, the maximal initial cefalexin concentration did not exceed 10 mg l^−1^ [21,22]. But in this study, the results of cefalexin degradation at different initial concentrations illustrated that both CLX-2 and CLX-3 were able to tolerate high concentrations of cefalexin even at the concentration of 100 mg l^−1^ (figure 9). The degradation patterns of cefalexin by two strains under different initial concentrations were similar and CLX-3 has a relatively higher degradation efficiency. Normally, the degradation efficiency of pollutants is related to their initial concentrations [37,38]. However, the results in this study demonstrated that the increase in initial concentration did not lower the removal rates of cefalexin, which was consistent with the result of cefalexin degradation by *Pseudomonas sp.* CE22 in another study [21]. Shi *et al*. found that an increase in initial concentration (25–150 mg l^−1^) of another tetracycline antibiotic, oxytetracycline, did not make a significant difference to its biodegradation efficiency by strain *Arthrobacter nicotianae* OTC-16 [39]. However, it has been proven that the lincomycin degradation rate by *Clostridium* sp. strain LCM-B was lowered by high initial concentrations in the range of 100–500 mg l^−1^ [38], suggesting different kinds of antibiotic-resistant bacteria can tolerate antibiotics with different concentrations. Although CLX-3 had a higher degradation rate, more than 99% of 100 mg l^−1^ cefalexin could be removed by both strains within 10 h, suggesting the potential of rapid cefalexin degradation at a high initial concentration by these two strains.

The processes of cefalexin biodegradation at different initial concentrations were also fitted with first order kinetic model (table 3). It was observed that with the increase of initial concentrations, the constant k also bulked, and the half-lives of cefalexin fell off, indicating the increase of the degradation rates. The *R*^2^ values at different initial concentrations ranged from 0.84 to 0.91 for *Rhizobium* sp. (CLX-2) and from 0.76 to 0.90 for *Klebsiella* sp. (CLX-3).

**Table 3.** First-order kinetic model of cefalexin degradation by CLX-2 and CLX-3 at different initial concentrations.

initial concentration (mg l^−1^)	equation	*k* (h^−1^)	*T*_1/2_(h)	*R*^2^
*Rhizobium* sp. (CLX-2)				
5	ln (*C*/*C_0_*) = −0.2576t	0.2576	2.69	0.84
10	ln (*C*/*C*_0_) = −0.2495t	0.2495	2.78	0.86
20	ln (*C*/*C*_0_) = −0.3685t	0.3685	1.88	0.88
50	ln (*C*/*C*_0_) = −0.5435t	0.5435	1.28	0.91
100	ln (*C*/*C*_0_) = −0.5934t	0.5934	1.17	0.90
*Klebsiella* sp. (CLX-3)				
5	ln (*C*/*C*_0_) = −0.1688t	0.1688	4.11	0.85
10	ln (*C*/*C*_0_) = −0.2948t	0.2948	2.35	0.76
20	ln (*C*/*C*_0_) = −0.2321t	0.2321	2.99	0.78
50	ln (*C*/*C*_0_) = −0.3758t	0.3758	1.84	0.88
100	ln (*C*/*C*_0_) = −0.4162t	0.4162	1.67	0.90

### 3.4. Identification of metabolites and proposed pathway

**Figure 10.**
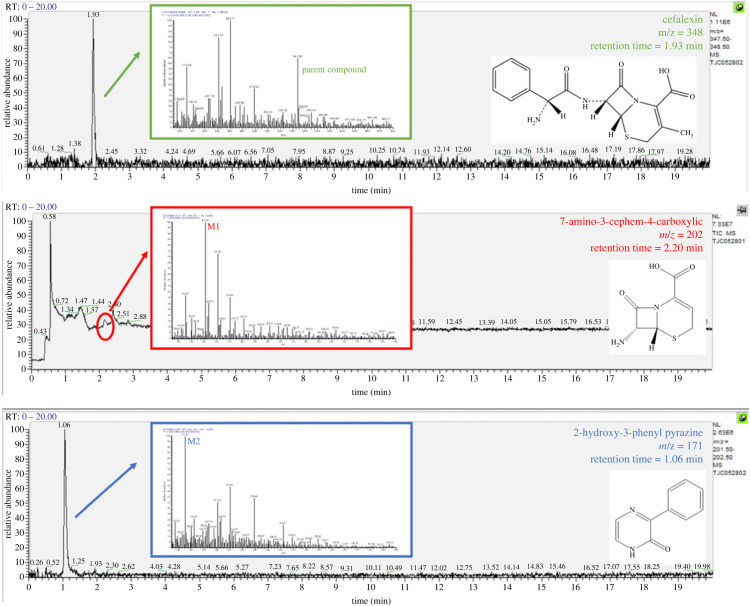
Mass spectra of major cefalexin degradation metabolites.

The mass spectra during cefalexin biodegradation by *Klebsiella* sp. (CLX-3) were recorded. The fragment with a *m/z* of 348 confirmed the presence of the parent compound, cefalexin. Two major metabolites with molecular ions at *m*/*z* 202 and 171 were identified under full scan mode and designated as intermediate M1 and M2, respectively (figure 10). The metabolites M1 and M2 were identified to be 7-amino-3-cephem-4-carboxylic acid and 2-hydroxy-3-phenyl pyrazine, respectively, based on their corresponding *m/z* value of molecular ions, compound structures and chemical bond composition of cefalexin.

According to previous studies, 2-hydroxy-3-phenyl-6-methylpyrazine has been detected as an acidic degradation product of cephalexin in the presence of formaldehyde [40]. During the degradation process of another *β*-lactam antibiotic, cefaclor, 2-hydroxy-3-phenyl pyrazine has also been reported as a major product under a high temperature (85°C) [41]. The formation of 2-amino-2-phenylacetamide was detected in an electrochemical degradation process of cefalexin [16], which may be the precursor of the metabolite 2-hydroxy-3-phenyl in this study. Lin *et al*. [21] found two *Pseudomonas* strains capable of degrading cefalexin and 2-hydroxy-3-phenyl pyrazine was identified during their degradation, whose result accorded with our current finding of 2-hydroxy-3-phenyl as a biodegradation intermediate of cefalexin by bacteria. However, to the best of our knowledge, this study was the first to report the identification of 7-amino-3-cephem-4-carboxylic acid during the biodegradation process of cefalexin. Therefore, it could be inferred that cefalexin degradation by *Klebsiella* sp. (CLX-3) first occurred via hydrolysis of the peptide bond to give rise to 7-amino-3-cephem-4-carboxylic acid and 2-amino-2-phenylacetamide, then intramolecular attack of the primary amine of the side chain occurred in 2-amino-2-phenylacetamide and 2-hydroxy-3-phenyl was subsequently generated. Due to the fact that the same peak with a *m/z* value of 202 was not detected at 24 h, it is possible that 7-amino-3-cephem-4-carboxylic was degraded to smaller compounds via hydrolysis of the four-membered *β*-lactam ring. However, smaller compounds were not captured since the mass range of UHPLC–MS spectra was 150–500. Therefore, further research still needs to be conducted for more precise determination of the specific biodegradation products and pathway.

## 4. Conclusion

Two bacterial strains (*Rhizobium* sp. (CLX-2) and *Klebsiella* sp. (CLX-3)) capable of degrading cefalexin were isolated from the activated sludge. They were able to degrade over 99% of cefalexin at an initial concentration of 10 mg l^−1^ within 12 h. The environmental conditions including temperature, pH, and inoculation dosage had significant influences on the degradation efficiencies of cefalexin by the two strains, while the initial cefalexin concentration did not exhibit significant effects. 7-amino-3-cephem-4-carboxylic acid and 2-hydroxy-3-phenyl pyrazine were identified as major metabolites during cefalexin degradation by *Klebsiella* sp. (CLX-3). This study provides a possible approach to bioremediation of cefalexin-polluted sites by bacterial strains. It is worth noting that *Rhizobium* is not a typical strain of activated sludge, and that knowledge about abundance and activity in sludge and in the used sludge is not given in this study. The protocol of strain isolation may give a preferential selection for the strain. For further work on these strains in sludge, it should be important to quantify *Rhizobia* and *Klebsiella* populations in activated sludge and to deduce from the population numbers if the strains may be used for augmentation.

## Data Availability

The datasets supporting this article are available from the Dryad Digital Repository: https://doi.org/10.5061/dryad.37pvmcvn4 [42].

## References

[1] Zhang QQ, Ying GG, Pan CG, Liu YS, Zhao JL. 2015 Comprehensive evaluation of antibiotics emission and fate in the river basins of China: source analysis, multimedia modeling, and linkage to bacterial resistance. Environ. Sci. Technol. **49**, 6772-6782. (10.1021/acs.est.5b00729) 25961663

[2] Cheng D, Liu X, Wang L, Gong W, Liu G, Fu W, Cheng M. 2014 Seasonal variation and sediment-water exchange of antibiotics in a shallower large lake in North China. Sci. Total Environ*.* **476–477**, 266-275. (10.1016/j.scitotenv.2014.01.010) 24468501

[3] Wang B, Yan J, Li G, Zhang J, Zhang L, Li Z, Chen H. 2020 Risk of penicillin fermentation dreg: increase of antibiotic resistance genes after soil discharge. Environ. Pollut. **259**, 113956. (10.1016/j.envpol.2020.113956) 32023801

[4] Sarmah AK, Meyer MT, Boxall ABA. 2006 A global perspective on the use, sales, exposure pathways, occurrence, fate and effects of veterinary antibiotics (VAs) in the environment. Chemosphere **65**, 725-759. (10.1016/j.chemosphere.2006.03.026) 16677683

[5] Zorita S, Mårtensson L, Mathiasson L. 2009 Occurrence and removal of pharmaceuticals in a municipal sewage treatment system in the south of Sweden. Sci. Total Environ. **407**, 2760-2770. (10.1016/j.scitotenv.2008.12.030) 19157523

[6] Lindberg R, Jarnheimer PÅ, Olsen B, Johansson M, Tysklind M. 2004 Determination of antibiotic substances in hospital sewage water using solid phase extraction and liquid chromatography/mass spectrometry and group analogue internal standards. Chemosphere **57**, 1479-1488. (10.1016/j.chemosphere.2004.09.015) 15519392

[7] Gao L, Shi Y, Li W, Niu H, Liu J, Cai Y. 2012 Occurrence of antibiotics in eight sewage treatment plants in Beijing, China. Chemosphere **86**, 665-671. (10.1016/j.chemosphere.2011.11.019) 22154158

[8] Lindsey ME, Meyer M, Thurman EM. 2001 Analysis of trace levels of sulfonamide and tetracycline antimicrobials in groundwater and surface water using solid-phase extraction and liquid chromatography/mass spectrometry. Anal. Chem. **73**, 4640-4646. (10.1021/ac010514w) 11605842

[9] Kim SC, Carlson K. 2007 Temporal and spatial trends in the occurrence of human and veterinary antibiotics in aqueous and river sediment matrices. Environ. Sci. Technol. **41**, 50-57. (10.1021/es060737+) 17265926

[10] Ledezma Estrada A, Li YY, Wang A. 2012 Biodegradability enhancement of wastewater containing cefalexin by means of the electro-Fenton oxidation process. J. Hazard. Mater*.* **227-228**, 41-48. (10.1016/j.jhazmat.2012.04.079) 22664258

[11] Homem V, Santos L. 2011 Degradation and removal methods of antibiotics from aqueous matrices - a review. J. Environ. Manage. **92**, 2304-2347. (10.1016/j.jenvman.2011.05.023) 21680081

[12] Peng JJ, Balasubramanian B, Ming YY, Niu JL, Yi CM, Ma Y, Liu WC. 2021 Identification of antimicrobial resistance genes and drug resistance analysis of *Escherichia coli* in the animal farm environment. J. Infect. Public Health **14**, 1788-1795. (10.1016/j.jiph.2021.10.025) 34785168

[13] Watkinson AJ, Murby EJ, Costanzo SD. 2007 Removal of antibiotics in conventional and advanced wastewater treatment: Implications for environmental discharge and wastewater recycling. Water Res. **41**, 4164-4176. (10.1016/j.watres.2007.04.005) 17524445

[14] Minh TB et al. 2009 Antibiotics in the Hong Kong metropolitan area: Ubiquitous distribution and fate in Victoria Harbour. Mar. Pollut. Bull. **58**, 1052-1062. (10.1016/j.marpolbul.2009.02.004) 19268314

[15] Zhang Y, Wang A, Ren S, Wen Z, Tian X, Li D, Li J. 2019 Effect of surface properties of activated carbon fiber cathode on mineralization of antibiotic cefalexin by electro-Fenton and photoelectro-Fenton treatments: mineralization, kinetics and oxidation products. Chemosphere **221**, 423-432. (10.1016/j.chemosphere.2019.01.016) 30648647

[16] Wang Q, Tu S, Wang W, Chen W, Duan X, Chang L. 2021 Optimized Indium modified Ti/PbO2 anode for electrochemical degradation of antibiotic cefalexin in aqueous solutions. Colloids Surfaces A Physicochem. Eng. Asp. **628**, 127244. (10.1016/j.colsurfa.2021.127244)

[17] Qian Y, Xue G, Chen J, Luo J, Zhou X, Gao P, Wang Q. 2018 Oxidation of cefalexin by thermally activated persulfate: kinetics, products, and antibacterial activity change. J. Hazard. Mater. **354**, 153-160. (10.1016/j.jhazmat.2018.05.004) 29751171

[18] Zhi D, Yang D, Zheng Y, Yang Y, He Y, Luo L, Zhou Y. 2019 Current progress in the adsorption, transport and biodegradation of antibiotics in soil. J. Environ. Manage. **251**, 109598. (10.1016/j.jenvman.2019.109598) 31563054

[19] Pan L, Li J, Li C, Tang X, Yu G, Wang Y. 2018 Study of ciprofloxacin biodegradation by a Thermus sp. isolated from pharmaceutical sludge. J. Hazard. Mater. **343**, 59-67. (10.1016/j.jhazmat.2017.09.009) 28941838

[20] Shao S, Hu Y, Cheng J, Chen Y. 2018 Degradation of oxytetracycline (OTC) and nitrogen conversion characteristics using a novel strain. Chem. Eng. J. **354**, 758-766. (10.1016/j.cej.2018.08.032)

[21] Lin B, Lyu J, Lyu X, Yu H, Hu Z, Lam JCW, Lam PKS. 2015 Characterization of cefalexin degradation capabilities of two Pseudomonas strains isolated from activated sludge. J. Hazard. Mater. **282**, 158-164. (10.1016/j.jhazmat.2014.06.080) 25070321

[22] Liu H, Yang Y, Ge Y, Zhao L, Long S, Zhang R. 2016 Interaction between common antibiotics and a Shewanella strain isolated from an enhanced biological phosphorus removal activated sludge system. Bioresour. Technol. **222**, 114-122. (10.1016/j.biortech.2016.09.096) 27716563

[23] Peng J, Wang X, Yin F, Xu G. 2019 Characterizing the removal routes of seven pharmaceuticals in the activated sludge process. Sci. Total Environ. **650**, 2437-2445. (10.1016/j.scitotenv.2018.10.004) 30292999

[24] Terzic S, Udikovic-Kolic N, Jurina T, Krizman-Matasic I, Senta I, Mihaljevic I, Loncar J, Smital T, Ahel M. 2018 Biotransformation of macrolide antibiotics using enriched activated sludge culture: kinetics, transformation routes and ecotoxicological evaluation. J. Hazard. Mater. **349**, 143-152. (10.1016/j.jhazmat.2018.01.055) 29414746

[25] Zhang Q, Liu H, Saleem M, Wang C. 2019 Biotransformation of chlorothalonil by strain Stenotrophomonas acidaminiphila BJ1 isolated from farmland soil. R. Soc. Open Sci. **6**, 1-9. (10.1098/rsos.190562) PMC6894561 31827822

[26] Heydari L, Bayat H, Gregory AS. 2021 Investigating the effect of inoculation of chickpea with rhizobium and mycorrhizal fungi (Funneliformis mosseae) on soil mechanical and physical behavior. Geoderma **385**, 114860. (10.1016/j.geoderma.2020.114860)

[27] Chen S, Zhang K, Jha RK, Chen C, Yu H, Liu Y, Ma L. 2019 Isotope fractionation in atrazine degradation reveals rate-limiting, energy-dependent transport across the cell membrane of gram-negative rhizobium sp. CX-Z. Environ. Pollut. **248**, 857-864. (10.1016/j.envpol.2019.02.078) 30856501

[28] Yessica GP, Alejandro A, Ronald FC, José AJ, Esperanza MR, Samuel CSJ, Remedios MLMA, Ormeño-Orrillo E. 2013 Tolerance, growth and degradation of phenanthrene and benzo[a]pyrene by Rhizobium tropici CIAT 899 in liquid culture medium. Appl. Soil Ecol. **63**, 105-111. (10.1016/j.apsoil.2012.09.010)

[29] Chen BY, Chen WM, Chang JS. 2007 Optimal biostimulation strategy for phenol degradation with indigenous rhizobium Ralstonia taiwanensis. J. Hazard. Mater. **139**, 232-237. (10.1016/j.jhazmat.2006.06.022) 16844294

[30] Shao S, Hu Y, Cheng C, Cheng J, Chen Y. 2018 Simultaneous degradation of tetracycline and denitrification by a novel bacterium, Klebsiella sp. SQY5. Chemosphere **209**, 35-43. (10.1016/j.chemosphere.2018.06.093) 29913397

[31] Zhang B, Wang M, Qu J, Zhang Y, Liu H. 2021 Characterization and mechanism analysis of tylosin biodegradation and simultaneous ammonia nitrogen removal with strain Klebsiella pneumoniae TN-1. Bioresour. Technol. **336**, 125342. (10.1016/j.biortech.2021.125342) 34082338

[32] Selvi A, Salam JA, Das N. 2014 Biodegradation of cefdinir by a novel yeast strain, Ustilago sp. SMN03 isolated from pharmaceutical wastewater. World J. Microbiol. Biotechnol **30**, 2839-2850. (10.1007/s11274-014-1710-4) 25086584

[33] Ali SW, Li R, Zhou W, Sun J, Guo P, Ma J, Li S. 2010 Isolation and characterization of an abamectin-degrading Burkholderia cepacia-like GB-01 strain. Biodegradation **21**, 441-452. (10.1007/s10532-009-9314-7) 19937266

[34] Wang YS, Zheng XC, Hu QW, Zheng YG. 2015 Degradation of abamectin by newly isolated Stenotrophomonas maltophilia ZJB-14120 and characterization of its abamectin-tolerance mechanism. Res. Microbiol. **166**, 408-418. (10.1016/j.resmic.2015.04.002) 25957243

[35] Huang M, Tian S, Chen D, Zhang W, Wu J, Chen L. 2012 Removal of sulfamethazine antibiotics by aerobic sludge and an isolated Achromobacter sp. S-3. J. Environ. Sci. (China) **24**, 1594-1599. (10.1016/S1001-0742(11)60973-X) 23520866

[36] Peng X, Qu X, Luo W, Jia X. 2014 Co-metabolic degradation of tetrabromobisphenol A by novel strains of Pseudomonas sp. and Streptococcus sp. Bioresour. Technol. **169**, 271-276. (10.1016/j.biortech.2014.07.002) 25062538

[37] Daniel Elcey C, Mohammad Kunhi AA. 2010 Substantially enhanced degradation of hexachlorocyclohexane isomers by a microbial consortium on acclimation. J. Agric. Food Chem. **58**, 1046-1054. (10.1021/jf9038259) 20041660

[38] Wang M, Cai C, Zhang B, Liu H. 2018 Characterization and mechanism analysis of lincomycin biodegradation with Clostridium sp. strain LCM-B isolated from lincomycin mycelial residue (LMR). Chemosphere **193**, 611-617. (10.1016/j.chemosphere.2017.11.055) 29169137

[39] Shi Y, Lin H, Ma J, Zhu R, Sun W, Lin X, Zhang J, Zheng H, Zhang X. 2021 Degradation of tetracycline antibiotics by Arthrobacter nicotianae OTC-16. J. Hazard. Mater. **403**, 123996. (10.1016/j.jhazmat.2020.123996) 33265032

[40] Nuñez-Vergara LJ, Squella JA, Silva MM. 1982 Polarography of an acidic degradation product from cephalexin. Talanta **29**, 137-138. (10.1016/0039-9140(82)80036-2) 18963099

[41] Dorman DE, Lorenz LJ, Occolowitz JL, Spangle LA, Collins MW, Bashore FN, Baertschi SW. 1997 Isolation and structure elucidation of the major degradation products of cefaclor in the solid state. J. Pharm. Sci. **86**, 540-549. (10.1021/js960428p) 9145376

[42] Tian J, Chen C, Lartey-Young G, Ma L. 2022 Data from: Data of cefalexin degradation, bacterial growth and effects of different environmental factors. Dryad Digital Repository. (10.5061/dryad.37pvmcvn4)

